# Routine developmental screening in Australian general practice: a pilot study

**DOI:** 10.1186/s12875-023-02093-7

**Published:** 2023-07-10

**Authors:** Karyn Alexander, Danielle Mazza

**Affiliations:** Department of General Practice, Monash University, 553 St Kilda Road, Melbourne, VIC 3004 Australia

**Keywords:** Developmental screening, Developmental surveillance, Child health, General Practice, Team care

## Abstract

**Background:**

Parents’ Evaluation of Developmental Status, (PEDS), is a validated screening tool designed for primary health care clinicians to assess child development. Despite widespread use by local government child-nurse services, PEDS has not been tested in Australian general practice. We examined the effect of an intervention that aimed to use PEDS to improve documented assessment of child developmental status during routine general practice consultations.

**Methods:**

The study took place in a single general practice in Melbourne, Australia. The intervention included training of all general practice staff regarding PEDS processes and provision of PEDS questionnaires, scoring and interpretation forms. Mixed methods incorporated audits of clinical records of young children (1 to ≤ 5 years) before and after the intervention, and written questionnaires and a focus group (informed by the Theoretical Domains Framework and COM-B model) with receptionists, practice nurses and general practitioners.

**Results:**

Documented developmental status more than doubled after the intervention with almost one in three (30.4%) records documenting the PEDS tool. Overall, staff responses to questionnaires indicated that PEDS processes had been successfully implemented, half of the staff felt PEDS had developed their professional skills and clinicians expressed confidence using the tool (71%). Thematic analysis of the focus group transcript revealed divided reactions to PEDS screening with most barriers arising from general practitioners’ motivation to use PEDS tools and perceptions of environmental constraints.

**Conclusions:**

A team-practice intervention that applied PEDS training and implementation, more than doubled documented rates of child developmental status during routine visits. Solutions to underlying barriers could be incorporated into a revised training module. Future studies need to test the tool in more methodologically robust studies that include analysis of the outcomes of developmental surveillance and long-term sustainability of PEDS use in practices.

**Supplementary Information:**

The online version contains supplementary material available at 10.1186/s12875-023-02093-7.

## Background

**Fig. 1:**
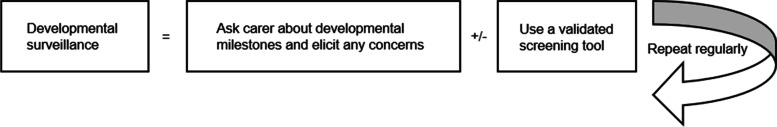
Model to define child developmental surveillance

In countries like Australia, developmental disability, behavioural and mental health disorders impact between 10 and 20 percent of young children [1, 2] but often present too late for effective early intervention [3, 4]. General practice guidelines recommend opportunistic and proactive preventive healthcare with children [5] to supplement developmental surveillance programs offered by Child and Family Health Nursing (CFHN), specialist nursing services that operate outside of general practice. Despite CFHN services being free at the point of care, population coverage is less than 50 percent in some states, [6] leaving opportunities for general practitioners (GPs) to review preventive health measures and assess child development during minor illness visits. However, time constraints and lack of knowledge of child developmental screening instruments are barriers to GP-led developmental surveillance (Fig. 1) [7].

One solution is to utilise validated screening instruments that replace direct clinical assessment with accurate recording and interpretation of ***parental observations*** of child development, thus saving practitioners’ time [8]. If a parent expresses a concern about their child’s development, there is a high probability that there ***is*** a problem [9, 10]. Parents’ Evaluation of Developmental Status (PEDS), a parent questionnaire of child health and development, can be completed in under two minutes (whilst parents wait in the waiting room) and can be rapidly scored and interpreted by clinicians (Table 1) to categorise children according to low-, medium-, and high risk of developmental disability [11]. PEDS has a reported sensitivity of (91–97 percent) and specificity of (73–86 percent) [12].

**Table 1:** Summary of content of Parents’ Evaluation of Developmental Status (PEDS) [11]

Eight questions that elicit parent concerns in developmental domains of:
• Receptive language
• Fine motor
• Gross motor
• Behaviour
• Expressive language
• Social-emotional
• Self-help
• School / pre-school skills
And two open ended questions

PEDS was designed for use in primary care and is already widely applied by CFHN [13]. Although recommended by GP-guidelines [5], PEDS has not been adequately tested in Australian general practice and it is not known if general practice nurses (PNs) or GPs would find it feasible and acceptable to incorporate PEDS into typical child health consultations.

## Methods

### Aims

This study aimed to determine whether PEDS in general practice was feasible and acceptable, and could increase the documentation of child developmental status, using a PEDS-training and implementation intervention. Ethics approval was obtained from the Monash University Human Research Ethics Committee and all participants provided informed consent.

### Setting and participants

**Fig. 2:**
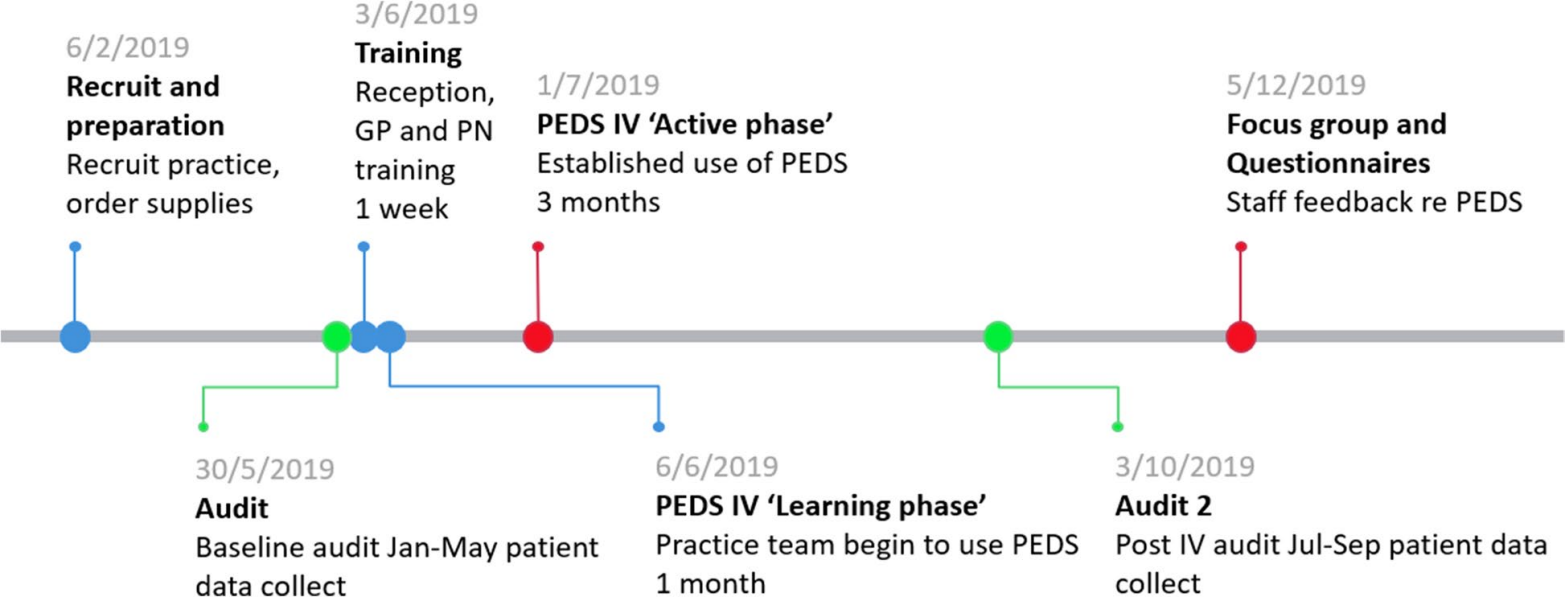
Timeline for the Parents’ Evaluation of Developmental Status (PEDS) intervention study

A moderately sized general practice clinic (11 GPs, 3 PNs) was recruited to the study after the GP-owner expressed interest in participating following an open invitation at a GP conference (Fig. 2). The practice is located in a socioeconomically disadvantaged suburb south-east of Melbourne, Australia, home to a greater proportion of children under 18 years [14]. At the time of this study in this practice, routine appointments and vaccinations did not incur out of pocket expenses for citizens and permanent residents (Medicare Australia card-holders).

### Intervention

**Fig. 3:**
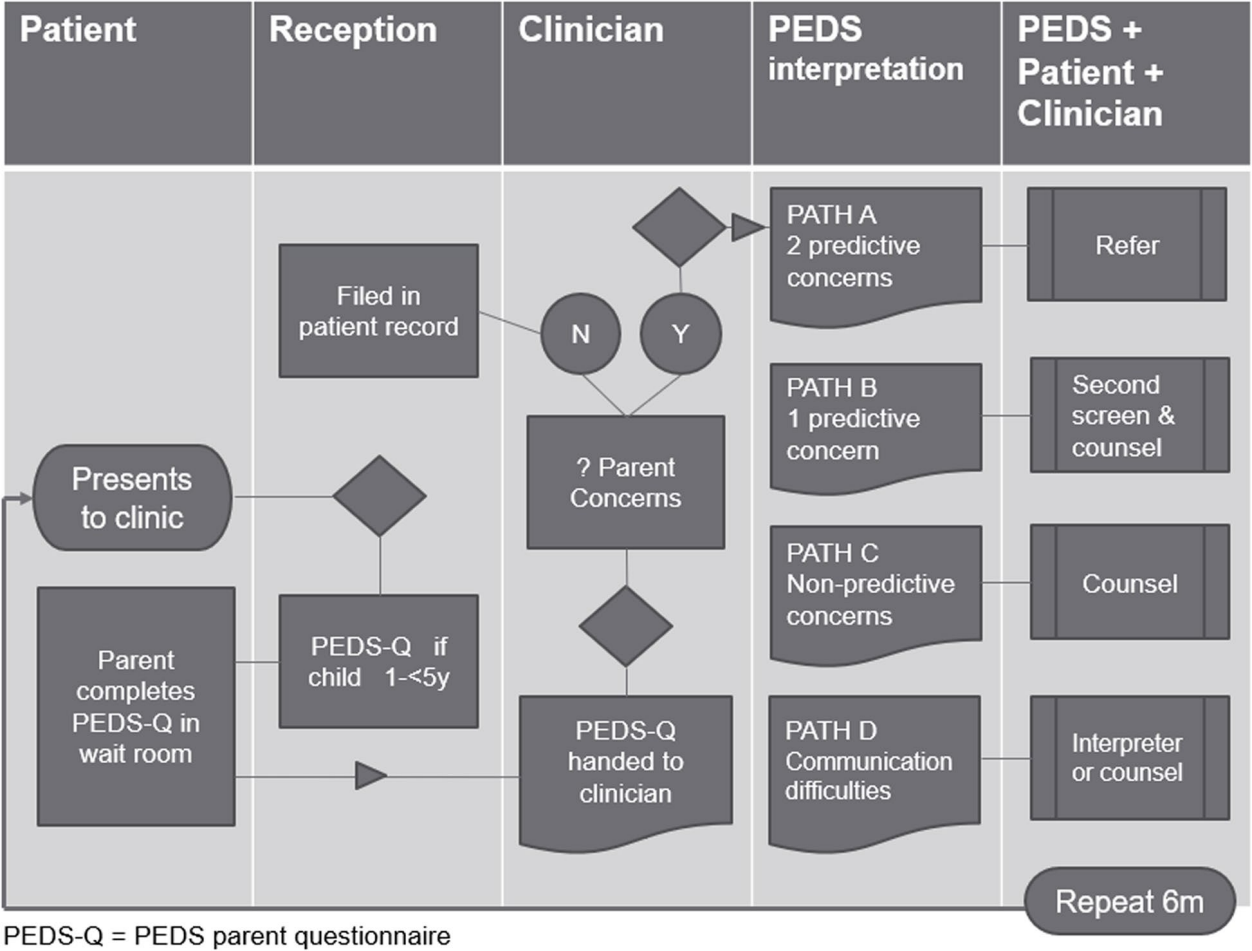
Parents’ Evaluation of Developmental Status (PEDS) processes and decision points (◊)

The practice was provided with all PEDS resources including questionnaires, score sheets and interpretation forms. The “PEDS: Developmental Milestones (PEDS:DM) Complete Kit” was provided as an additional resource to enable practitioners to assess development through direct testing (where a concern requires further evaluation). Parent tip-sheets regarding management of preschool behavioural problems were also provided in electronic format. All clinic staff were offered one hour of PEDS-training (over lunchbreak, with lunch provided) in one of three sessions according to their role in the practice (Receptionist, PN, GP). Training was provided by one of the researchers (KA), an accredited PEDS trainer. Reception staff were provided with PEDS questionnaires, trained to identify age-eligible children (aged 1- ≤ 5 years) and role-played what to say when handing questionnaires to parents. GPs and PNs learned how to transcribe completed parent questionnaires onto the score sheet and interpret scores according to risk for developmental delays. Scores determine whether to refer, counsel, rescreen or monitor development (Fig. 3).

### Data collection

#### Questionnaires

At the end of the study period, all staff were requested to complete questionnaires regarding PEDS implementation and child developmental surveillance: Four questions asked for views on the study, use of PEDs, adapting to new tasks and levels of comfort using PEDS. Three questions asked for views on child developmental assessment in general, who should do it and when it should be done. Five questions (clinicians only) asked for rating of knowledge about early intervention services and confidence recalling developmental milestones, detecting developmental problems and red flags for Autism in young children. Surveys, adapted from previous research studies in preventive healthcare for children, [15, 16] have been published in a previous study (Table 2) [17].

**Table 2:** Clinician and receptionist responses to questionnaire regarding Parents’ Evaluation of Developmental Status (PEDS) implementation and documented developmental concerns

Questions 1–7: all staff *(n* = *8)*, 8–12: clinicians only *(n* = *7)*	Strongly disagree	Disagree	No opinion	Agree	Strongly agree
1.This practice has successfully implemented PEDS screening with routine childhood visits			2	5	1
2.This practice has easily adapted to taking on new tasks and roles			2	6	
3. I have developed my professional skills working with young children			4	4	
4. I rate good personal levels of comfort asking parents to complete questionnaires about their child’s development			2	3	3
5. I believe Child and Family Health Nurses should be the main body responsible for assessing the development of pre-school children		2	2	4	
6. I believe pre-school children should have their development assessed in general practice only during vaccination appointments (when child is well)	1	3	2	2	
7. I believe pre-school children should have their development assessed in general practice at every available opportunity			2	3	3
8. I have good knowledge regarding how to access early intervention services for young children			2	4	1
9. I feel confident in my ability to detect developmental problems		1		5	1
10. I feel confident in my ability to use PEDS to help detect developmental problems in children aged 1–5 years			2	3	2
11. I feel confident in my recall of infant and child developmental milestones		2		2	3
12. I feel confident in my ability to detect the “red flags” for Autism in children aged 1–5 years		2		2	3

### Post-intervention staff focus group

All staff were invited to a single focus group, conducted during a lunch time, to further explore their experiences using PEDS and gather views about its potential impact on their practice, and referrals and outcomes for children.

### Audits

The electronic health records of all children aged 12 months to five years who had attended the clinic, in the five months before (baseline) and three months after the intervention, were extracted (to obtain a sample size of approximately 250 records per group, see Table 3). Audits recorded the proportions of records with documented evidence of developmental concerns and developmental status from:

- references to developmental concerns in health summaries of coded diagnoses, free text notes of the last consultation and consultations over preceding six months, letters from specialists and allied health.
- PEDS-questionnaires scanned into the record
- references to PEDS screening in consultation notes

**Table 3:** Sample size calculation (https://select-statistics.co.uk/calculators/sample-size-calculator-two-proportions/)

Based on an expected documentation of developmental status at a rate of 10% at baseline (in line with a baseline rate obtained in a previous study [17]) and aiming to double this to 20% following
the intervention [17], required a minimum sample of 197 records in each sample (given 95% confidence level and 80% power). Adding a 20% buffer
on this number (to allow for attrition by removing of duplicates) equates to 246 records, rounded to 250 per sample

Each ‘new’ behavioural concern was documented only once. Training, study oversight, clinical audit and analysis was conducted by an experienced GP-researcher who also led the focus group.

Audits were performed by the experienced researcher, working with three research assistants (two medical students and a junior doctor) who had each completed basic paediatric undergraduate training. They received additional training in early childhood development and PEDS processes, including how parents’ concerns are typically documented in the GP-electronic health record (Additional File 1. Audit processes, for keywords and coding details).

### Analysis

**Fig. 4:**
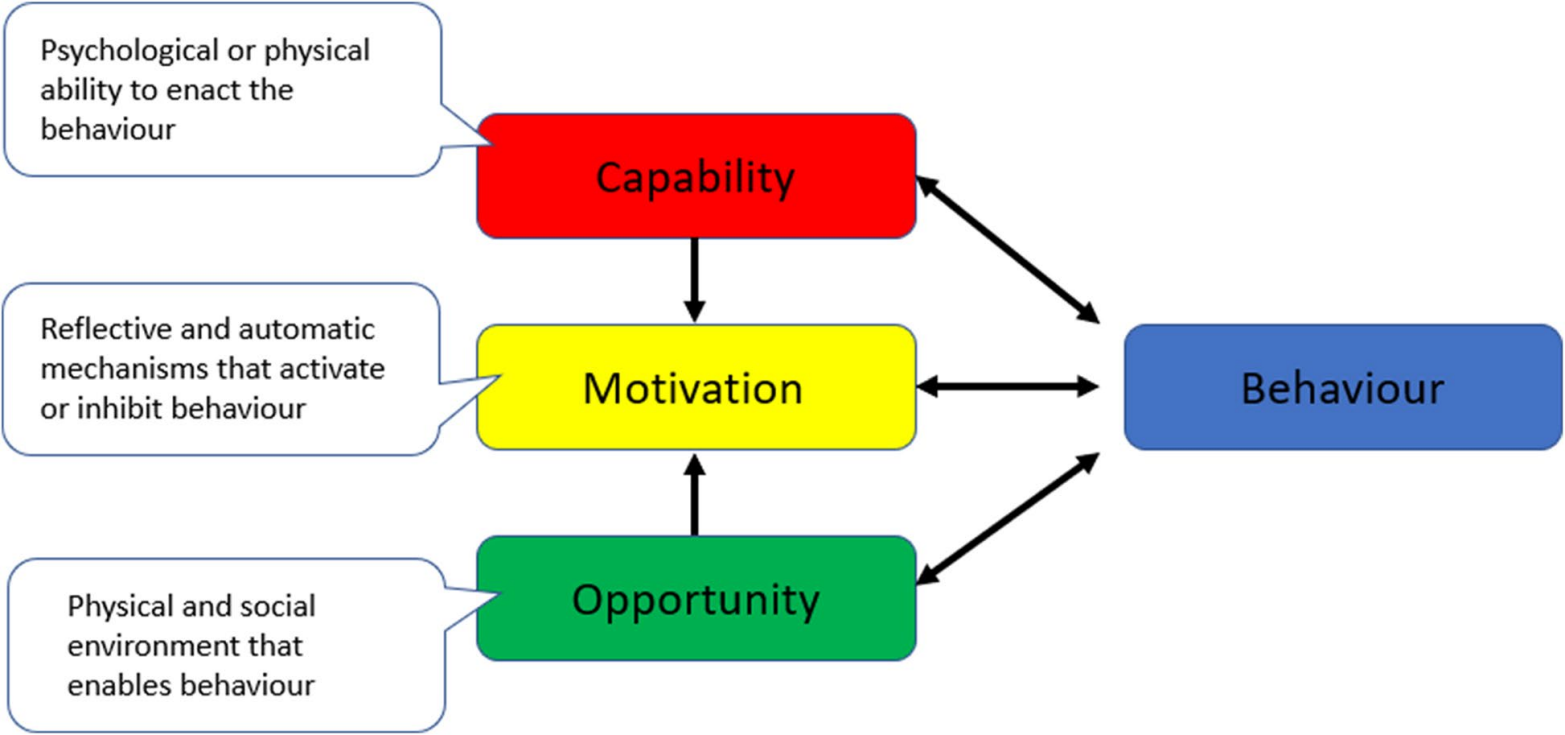
The COM-B model [18]

Qualitative data were recorded, transcribed, and thematically analysed utilising the Theoretical Domains Framework and COM-B model (Capability, Opportunity Motivation-Behaviour) [18], a framework used to understand how behaviour forms in context (Fig. 4).

## Results

Training was delivered to six (of 11 GPs), two (of three) PNs and two (of three) receptionists.

### Questionnaires

Questionnaires were completed by 7 of 14 (50%) eligible clinicians (six GPs, one PN) and one of two reception staff. One of these GPs had not received the PEDS-training. Six (75%) staff agreed PEDS processes had been successfully implemented, they had easily adapted to new tasks and roles and were comfortable asking parents to complete PEDS-questionnaires. Four (50%) staff felt that PEDS had developed their professional skills working with young children and five of seven (71.4%) clinicians expressed confidence using PEDS (Table 2).

GPs, PNs and reception staff held mixed beliefs about who should bear responsibility for child developmental surveillance: Three participants (37.5%) believed that CFHN should assess the development of young children, whilst two disagreed (25%) and three did not express an opinion. Two participants (25%) believed that assessment of child development in general practice should be confined to vaccination-visits, whilst six participants (75%) thought development should be assessed by GPs at ***every*** visit.

All except one clinician (PN) at this clinic expressed high levels of confidence detecting child developmental problems and most (five of seven, 71.4%) felt confident in their ability to recall developmental milestones and detect Autism spectrum disorders. The same proportion had good knowledge of how to access early intervention services (Table 2).

### Qualitative data

The focus group with 7 participants (1 Receptionist, 2 PNs and 4 GPs), all of whom had received training, provided additional insights into the PEDS implementation process, where it became apparent that beliefs and attitudes regarding PEDS were mixed. The practice had developed a novel way to use PEDS that differed from the process suggested during training that had PEDS questionnaires scored and interpreted at the time of the visit. In this practice, receptionists handed the questionnaires out to parents in the waiting room, from where they were passed to the practice nurse to score. If no concerns were identified the questionnaire was scanned into the record. If, however, a concern was identified, the questionnaire was placed with the completed scoresheet into the GP’s ‘administrative tray’, to be reviewed by the GP later-on. Parent concerns that were deemed ‘relevant’ were followed up either by the GP telephoning the parent to discuss a possible return visit or by annotating the file to address the concern at the next routine visit.

*“We … identified children in the age group; asked parents to fill in the form; once they filled in the form we checked if they marked any of the …any queries, any concerns, and then we passed them on to the nurses. Then filed it in their records.” (Receptionist).*

*“I did all the scoring and used the back of it and circled depending on what was found, the criteria, what the referral pathway was. So it made it really easy and from there it went on to the doctor”. (PN1).*

*“I’d look at the form if it was low-level issues, minor concerns, I’d probably make a note to discuss in the next consultation without specifically recalling someone for the purpose of doing that. If it was a more significant concern raised by the family or the score, I would ask them to come and make a routine appointment. To have a chat about it.” (GP2, male).*

The method adopted by the practice may have impacted the opinions formed about PEDS.

Capability (The psychological or physical ability to enact the behaviour)

The PN commented that since 2018, when Medicare rebated child health assessments in general practice were defunded, [19] she no longer saw children for health assessments and had felt de-skilled:

*“We were doing a lot [health assessments] but I had done a bit of extra education so I was quite confident, and now I just have no idea.” (PN1)*

The simplicity of PEDS helped her regain confidence:

“After a while it did become easier to figure out whether it’s a ‘Yes’ or a ‘No’. Initially I was a bit like, ‘Oh I’m not sure’” (PN1)

and now,

“I know how to score with my eyes closed …” (PN1)

GPs, however, felt they were quite knowledgeable about child development:

“You kind of have a rough idea … not … like when you went for exams, when you need to know exactly, you know. I think I’m quite comfortable, yes.” (GP4, female)

However, PEDS prompted them to consider the child’s development more often:

“It made me think more, in any consultation, a bit, … to be aware of it, to ask … which I did before but, I was a bit more, you know, now it’s more targeted.” (GP1, female)

Opportunity (The physical and social environmental barriers and enablers)

Some clinicians believed PEDS processes were straightforward and easily implemented,

“The major point is to do this flag, mark and have a look, it’s just excellent.” (GP1, female)

“It’s very easy to score and … the referral paths are very streamlined. It’s very easy to use” (PN1)whilst others believed they were onerous and, possibly, superfluous:

“It’s like anything, like with mental health. So I don’t think I need to go do a so-and-so questionnaire unless I need to, you know. I’ll ask the question around development without having a questionnaire, if there’s a concern raised in the consultation about development. I suppose it doesn’t come naturally to– when you are time-pushed anyway– to think ‘where’s that questionnaire’. Giving a questionnaire to someone could potentially take two minutes or it could take 10 minutes when you are waiting and you’re just sat there, you know. Whereas, when you are asking open questions about it you can get the information straight away.” (GP2, male)

Time constraints were a recurrent theme in this busy general practice:

“Some of the communities from Africa, I think there is an issue, they don’t understand it and they want us to fill it in and we’ve only got a 10-minute appointment so there’s no time to actually go through that with them.” (GP4, female)

GPs also highlighted a lack of referral pathways and treatment costs as barriers to developmental screening:

“The thing is the compliance in the long term. If there is any disability, we don’t have a clear cut referral pathway.” (GP3, male)

“I have been referring a few to [hospital name] but for some reason they are not accepting new referrals.” (GP4, female)

“And the speech [pathologist] is generally 150 dollars and you get 53 dollars back, the rebate is, so it’s still 100 out of pocket” (GP2, male)

Motivation (Reflective and automatic mechanisms that activate or inhibit behaviour)

When PEDS was first rolled-out the receptionist recalled feeling uncomfortable handing questionnaires to patients. This was quickly overcome, in part, through using a set ‘script’ as a reminder of what to say:

“It was more so, like, just remembering what to say because we kind of like had it scripted, then we got used to it, but then it became a bit of a routine.” (Receptionist)

One GP remained convinced the outcomes of PEDS made screening worthwhile:

“We have definitely identified about 30 kids we have to do something. I don’t think we would do that without this. Well, maybe, but we don’t have evidence of that … we’ve got some kids that we have to pay attention [to]… It’s a reasonable amount of, number of kids, finding them in the community. We’ve got to screen them. I am happy we can do that, so that is something.” (GP1, female)

### Audits

**Fig. 5:**
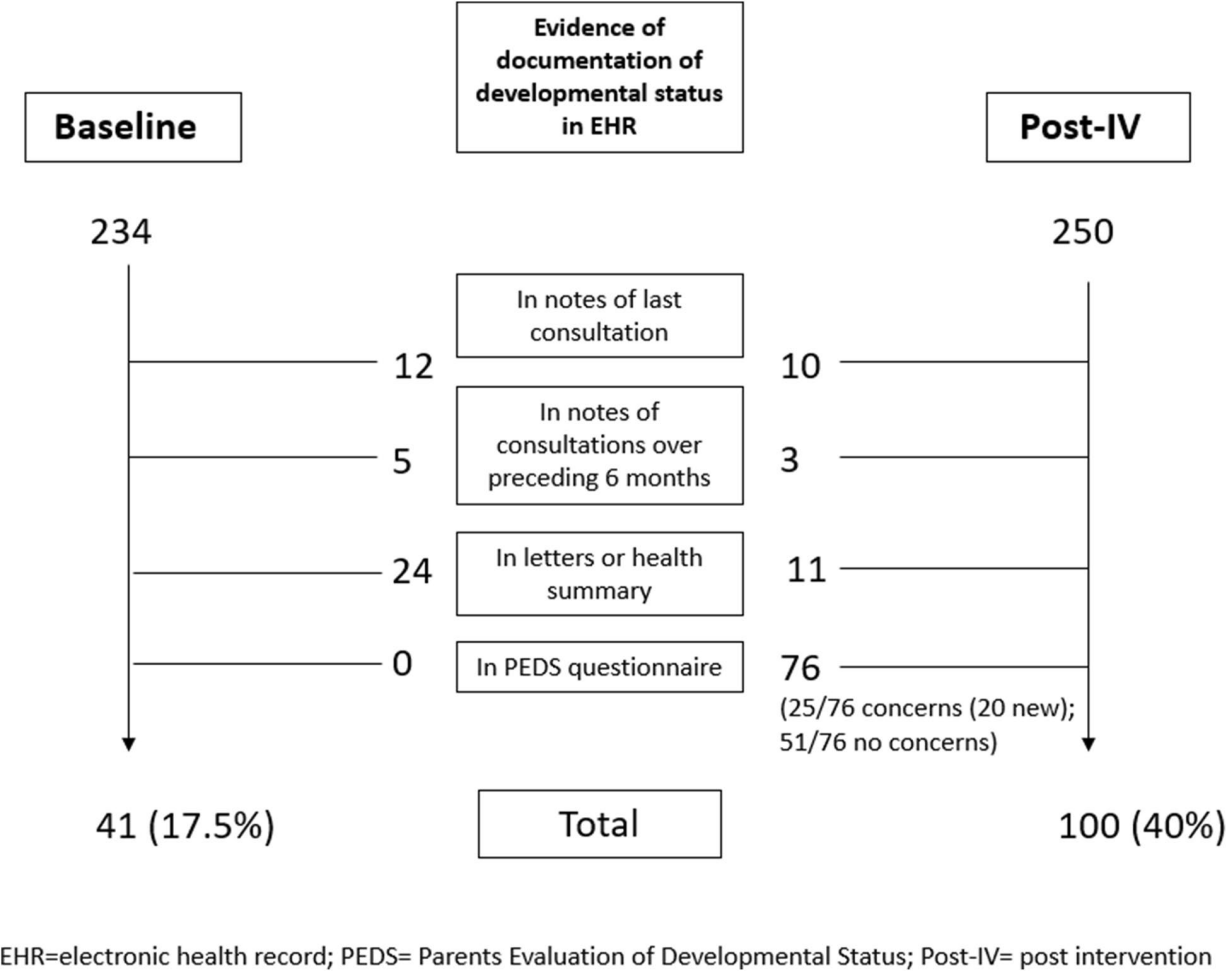
Audit of clinical records of children aged 1- ≤ 5 years, at baseline and post PEDS-intervention, showing evidence of documentation of developmental status

Documentation of developmental status – recorded PEDS, developmental assessment or parents’ developmental concerns– more than doubled (2.3 times) following the PEDS intervention (Fig. 5). A total of 486 child records of children aged 12 months to ≤ 5 years were analysed:

- 234 at baseline, consultations over five months, January-May 2019.
- 252 after intervention, consultations over three months, July–September 2019, reflecting increased consultation rates during Australia’s winter months

Two records were removed from the second data set because they had already been included in the baseline sample, leaving a total of 250 records. At baseline, PEDS was not documented in any of the clinical records, but 12 records demonstrated an aspect of child development had been considered during the last consultation, five recorded a developmental concern in consultation notes over the preceding six months and 24 recorded a developmental concern in the health summary or in correspondence. This represents a total of 41 (of 234) (17.5%) records documenting developmental status. Notably, at baseline no records indicated that development was assessed as a matter of routine, unless explicit parent concerns were raised. Following intervention, 76 records included a scanned copy, or documented, PEDS screening, with 25 of these documenting developmental concerns. Ten additional records recorded developmental status during the last consultation, three more recorded developmental status in consultations over the preceding six months and 11 more recorded developmental status in the health summary or correspondence. A total of 100 (of 250) (40%) records documented developmental status post intervention, an increase of 22.5%, (95% CI 14.7–30.26, P < 0.0001). In total, almost one in three (30.4%) consultations, post intervention, recorded PEDS and a quarter of these (20 of 76, 26.3%), recorded a parental concern for their child’s development not documented elsewhere (Fig. 5).

### PEDS analysis

In the post intervention sample, PEDS screening identified 25 (out of 250) children, 20 (8%) of whom this represented the first time a developmental concern was documented. Of the 37 developmental concerns noted: 13 had concerns about speech; ten had concerns about child behaviour, five had concerns about social and emotional health and three had gross-motor developmental concerns. Additionally, three noted general health concerns, and one hearing, one fine-motor and one self-help concern, were recorded (Table 4).

**Table 4:** Analysis of parental concerns in sample of 25 children with Parents’ Evaluation of Developmental Status (PEDS) documented in health record

	**PEDS domain**							
	Speech	Receptive language	Fine motor	Gross motor	Behaviour	Social-emotional	Self-help	Health
Single concern*	6	1	1	1	4	2		
more than 1 concern*	7			2	6	3	1	3
**Total**	**13**	**1**	**1**	**3**	**10**	**5**	**1**	**3**

Note: concern* = Parents’ concerns about their children’s development

### Researcher reflections

Reflections on PEDS training and processes by the researchers concluded that to accommodate the practice’s preferences for separate training of GPs, PNs and Reception staff, in addition to on-site data analysis taking longer than expected, the research burden substantially increased (eight visits in total). Reflections, supported by additional participant’ quotations are presented in Table 5.

**Table 5:** Lessons learned from Parents’ Evaluation of Developmental Status (PEDS) study

Researcher experience / Quote from Focus Group/ Barriers	Proposed solutions
Training time was more onerous than expected (three occasions over lunchtime) but designed to accommodate different roles and rosters GP-attendance was impacted by clinical demands	Reconsider delivery mode and consider protected time e.g. after hours
Audits were time-consuming, requiring multiple practice visits	Review natural word processing options for screening clinical records Ask practices to provide their own analysis
GP1: Drs did not show much of enthusiasm, I have to emphasise that, simply because not knowing much, not being really familiar with it, is a kind of being apprehensive it can increase work load	Present evidence of PEDS outcomes for children Provide further training re PEDS methodology, it should be streamlined into routine consultations according to staff role Include a business case for general practice
Referral pathways need to be streamlined and available	Workshop local referral pathways and present referral letter templates
PN: Initially I was a bit like, “Oh I’m not sure,” but then I –you sort of leave that in the hands of the doctors to decide what they want to do with it from there –as to whether or not it’s a true concern?	Provide more training to PNs to reduce GP workload and perceptions of burden
GP2: It’s like anything, like with mental health, so I don’t think I need to go do a so-and-so questionnaire unless I need to, you know	Present more evidence of PEDS outcomes for children Present more evidence of rates of child disability in area served by the practice
GP1: I don’t think routinely we’ll do it for every child unless we’ve got some concern	Emphasise PEDS can be routinely used for every child Place recall systems into health record e.g. every 6 months

## Discussion

This study demonstrated that training and implementation of PEDS, in a whole of practice intervention, was feasible and increased documentation and assessment of young children’s development. Documented developmental status more than doubled following the intervention and hinged on the receptionist handing out the PEDS questionnaire to parents in the waiting room (76 of 100 documentations of developmental status, post-intervention). PEDS was generally positively received and increased some clinicians’ skills and confidence detecting developmental delays in young children but was not appreciated by all GPs. Barriers to PEDS, revealed in the focus group discussion, included a lack of clear referral pathways, cost barriers for parents (specifically around speech therapy) and fears that using PEDS would increase the length of the consultation and doctors’ workloads more generally.

Our study is the first to report the use of PEDS during routine general practice consultations with children, in Australia, where there is already extensive uptake of PEDS by other primary care providers (CFHNs) [13]. Our previous study explored PEDS implementation with child vaccination consultations and reported similar findings [17], but this study has demonstrated that PEDS does not have to be confined to “well-child” (vaccine) visits.

Despite finding that the rate of documented developmental status more than doubled post-intervention, 70 percent of consultations did not have documented evidence of PEDS screening post intervention. Given that developmental delays affect more than one in five children [1, 2] and more subtle delays are easily overlooked, parents need to be actively and repeatedly encouraged to divulge any developmental concerns they may have about their children by all trained health professionals. Previous research underscores the need for a systemic approach to developmental surveillance: parents with low educational attainment are less likely to verbally express any developmental concerns they may have [20], and children who do not have documented developmental status recorded in their health record are more likely to be those children who ***experience*** developmental delays [13]. Comments from some clinicians in this study reflected reasons uncovered by other researchers that may explain a reluctance to participate in screening: an over-reliance on clinical impressions [21, 22] failure to recognise parents’ concerns [21, 23] and a lack of knowledge about the benefits of validated screening tools. [7, 24]. Garg et al. highlight the need for further training and recommend practice nurse support for GPs conducting developmental screening [7].

This practice developed a novel way to use PEDS that deviated from the pathway suggested during training and meant that parents’ concerns were not necessarily discussed at the time of the visit. This method may have placed an extra layer into PEDS-processes, increased doctors’ paperwork and perceptions of burden. PEDS-processes recommend scoring and discussion during the consultation: Acknowledging parents’ concerns at the time of consultation validates parents’ observations of their child and avoids the risk that some families could default on follow-up.

Nevertheless, time-pressures in Australian general practice may mean that clinicians prioritise healthcare according to clinical urgency. Time-pressures have also been raised as barriers to developmental screening by CFHNs [25] and were reported in our previous study that implemented PEDS only during vaccine visits [17]. In that study PNs commented that if something urgent came up they would put the score sheet aside to address later and, whilst influenza vaccine-clinics were operating, receptionists stopped handing out PEDS altogether [17]. It should be acknowledged that introducing PEDS will add time to some consultations, but the team approach–including the parent, receptionist and PN– dissipates some of the time burden, whilst retaining parent engagement.

In the United States (US), where clinicians report regular use of formal tools for developmental screening [26], PEDS has been widely applied for more than a decade and has some evidence for its utility [27]. The US context of ‘well child care’–preventative health consultations similar to those delivered by CFHNs in Australia– differs from this study’s application of PEDS to mild illness/routine GP consultations and may explain why rates documenting developmental concerns are reportedly much higher in US intervention studies [28]: A study that applied PEDS to well child care processes in two large urban primary care centres found almost two thirds of children (6 months to 8 years) documented developmental concerns during visits [29]. It also found that more behavioural and developmental concerns were detected using PEDS and generated more referrals [29]. Outcomes from our study were limited by small numbers of “positive” PEDS questionnaires, but our qualitative research, and that of others [7] suggests that clinicians can be motivated towards screening when they believe that referrals and clinical interventions will benefit the child. The corollary to this, voiced by some GPs in this study, is that they are demotivated when they believe there are insufficient services and pathways for referral to address the developmental concerns detected by screening- and, in this study, eight per cent of new ‘concerns” were detected directly through PEDS. This is an issue common to all new screening in general practice. GPs need to feel confident that the services available to patients are both sufficient and effective at managing the disorders they identify. The costs of seeking diagnostic and therapeutic services are additional barriers and have been acknowledged by previous Australian researchers [30].

Despite an average per-patient cost of approximately $2.50 US, online screening has been estimated to ultimately cost less than paper and pencil screens due to substantial savings in professionals’ time [31]. Research on smartphone applications and web-based innovations is limited but, apart from potential efficiencies, seems to be preferred by both caregivers and practitioners and may be equally effective [32]. In the US, “PEDStestOnline” includes PEDS, PEDS:DM and an Autism-specific screening tool. A parent-portal enables parents to complete tests before the visit with automated scores sent directly to clinics. A study reported on a sample of 22 (of 79) clinics using PEDStestOnline and found parents completing the test in the waiting room via a tablet or kiosk were more likely to complete screening than those asked to login at home via an appointment reminder card [31]. Smartphone PEDS tools have been reported in two additional exploratory studies conducted in South Africa, finding that community health workers using a smartphone version of a combined PEDS, PEDS:DM tool obtained similar outcomes to a health professional using paper versions of PEDS [33] and were overwhelmingly positive about the benefit of the screening program for their communities [34].

Regardless of the mode of delivery, developmental surveillance requires ‘buy in’ from practitioners. In this study, despite nine of 11 GPs consenting to the PEDS intervention, only six engaged with the project by responding to the questionnaire or actively participating in the focus group. Although participating in research is not the same as actual uptake of an intervention, ultimately PEDS processes cost the practice in terms of upskilling clinicians, the costs of purchasing copyrighted screening tools and their administration. Reimbursement through specific insurance-rebated services or block payments to practices for enhanced services [35] will require advocacy and time. In North Carolina, the “Assuring Better Child Health and Development (ABCD) Project” that aimed to *“assist practices in implementing an office process for screening”*, began in practices that self-selected (were motivated) to participate, but was readily adopted and, eventually, replicated state-wide [36]. Ultimately, the program influenced Medicaid policy, the US joint federal and state program that provides health coverage to low-income people.

The strengths of this study include the simplicity of PEDS that enabled it to be readily incorporated into practice processes, and the mixed methods approach that allowed exploration, analysis and a deeper understanding of how PEDS was implemented. There was, however, considerable burden placed upon researchers making multiple visits to the practice, fragmentation that possibly contributed to the ‘novel’ delivery of PEDS processes in this study. The intervention could be delivered to several practices (that ‘express an interest’) at a single convention and could request practices gather their own data. Such methods have been employed in US studies. We also need to be cautious about the extent our findings (measured over a short period, using a pre-post study design) would be generalisable across different general practice models in Australia. A longer follow-up period would be more desirable to ascertain whether ongoing support, training and quality improvement approaches would be required to sustain PEDS processes. Additional audits would be useful to ascertain levels of PEDS-use long term and further qualitative research could determine whether parents’ attitudes towards PEDS change with repeated exposure to questionnaires. The fact that a practice delivered training-intervention still only reached 10 of 17 potential participants indicates that further research regarding delivery of this intervention (Table 5) is required and could explore PEDS implementation across different general practice types (selected according to billing style, practice size and socio-demographics). There is an urgent need to research whether screening child-development in a general practice population of young children results in improved health-outcomes and whether clinical records adequately capture the potential outcomes from screening– detection, management and referral of child health and developmental problems. It should be noted that this study took place before telehealth became more broadly funded in Australia (during the Covid19 pandemic), which brings into question the viability of PEDS with telehealth consultations, another factor that would require additional research.

Arising from this and our previous study, we can recommend PEDS implementation as a valid qualitative improvement process. Our findings indicate that practitioner’ skills detecting developmental delay may benefit from PEDS training, and PNs seem particularly receptive to this. Expanding the use of common screening tools, like PEDS, could improve communication and understanding amongst healthcare professionals regarding child development, enabling earlier detection and intervention [37, 38]. Smartphone applications and web-based innovations are introducing new efficiencies to developmental surveillance using PEDS and other tools and seem to be preferred by both caregivers and practitioners [32–34].

## Conclusion

This study showed that PEDS is both feasible and, at least, partially acceptable and can increase clinicians’ confidence detecting developmental delays in young children. PEDS processes that included the receptionist and PN in the medical team more than doubled documented rates of child developmental status in this practice and should be further tested in a cluster randomised controlled trial that includes analysis of the outcomes of screening young children.

## Supplementary Information

**Additional file 1.**

## Data Availability

The datasets used and analysed during the current study are available from the corresponding author on reasonable request.

## Abbreviations

CFHN: Child and Family Health Nursing
GP: General Practitioner
PEDS: Parents’ Evaluation of Developmental Status
PN: Practice Nurse
