# Supplementary material for: Routine developmental screening in Australian general practice: a pilot study
Source: BMC Prim Care. 2023 Jul 10;24:143. doi: 10.1186/s12875-023-02093-7 (PMC10331965; doi:10.1186/s12875-023-02093-7)
Supplement: Supplementary file 1 — Additional file 1. [file 12875_2023_2093_MOESM1_ESM.docx]

Additional File 1. Audit processes

Inclusion criteria:

Child aged 12m to 4y 11m 30 days on the date of the last consultation

Examples of keywords for medical record review

| **Concern** | **Keywords** |
| --- | --- |
|  |  |
| Eyesight | Vision disorder/ amblyopia/ strabismus/ squint/ head-tilt |
| Hearing | Hearing disorders/ hearing loss/ selective hearing/ ignores/ |
| Eating habits | Picky eater/ decreased appetite/ eating disorder |
| Physical activity | Overactive/ Sedentary/ clumsy/ falls frequently |
| Speech and language development | Speech-language pathology/ not speaking/ language delay/ difficult to understand/ doesn’t understand speech delay/ stuttering/ lisp/ |
| Fine motor skills | pen-grip/ difficulty drawing simple shapes/ finds it hard to hold a pencil |
| Gross motor skills | Gait disorder/ walking-delayed/ sitting-delayed/ clumsy/ falls frequently/ can’t catch / kick ball |
| Behaviour and mood / Social and emotional | Autism spectrum disorder (ASD)/ Asperger’s syndrome/ attention deficit disorder (ADD) with hyperactivity (ADHD)/ over-active/ eye-contact (reduced)/ no pretend/imaginary-play/ behaviour* problems/ defiant disorder/ anxiety/ biting/ hitting/ tantrums/ temperament/ poor self-esteem/ whines/ depress*ed/ sibling rivalry/ attention seeking/ |
|  |  |
| Cognitive | Dis/interest in books/ reading/ numbers/ counting/ letters/ learning difficulty/ preschool problem |
| Other | “In their own world”; Abnormal play; Lazy/ seems lazy; Bullying/bullied; Loss of a skill; Parenting problem |

EXAMPLE of coding (not real data)

| Child identification  initials of first and last name | age | Date of last consultation  dd/mm/2019 | Consultation with PN or GP?  GP visit =1 PN visit =2 Both GP and PN =3 | Developmental  concern documented in record of last consultation  No=0  Yes=1 | Developmental  concern documented in records of consultations over preceding 6 months  No=0  Yes=1 | Domain of Developmental concern  0-9* | Past History of Developmental concern recorded in letters or summary more than 6 months before last consultation  No=0  Yes=1 | Domain of this Developmental concern  0-9* |
| --- | --- | --- | --- | --- | --- | --- | --- | --- |
| KA | 4y7m | 3/06/2019 | 1 | 1 | 0 | 1 | 0 | 0 |
| KH | 20m | 3/06/2019 | 2 | 0 | 1 | 4 | 1 | 9 |

*Codes for Domains of Developmental concern 0-9

0= not assessed

1= language/speech

2= eyesight/vision

3= hearing/receptive language

4= behavioural and sleep

5= physical-gross motor

6= physical-fine motor

7= social-emotional/mental health

8= cognitive/learning

9= other
